# Bibliography

**Published:** 1916-11

**Authors:** 


### BIBLIOGRAPHY

PEESO'S CROWN AND BRIDGE WORK. This new work has just been published from the house of Lea & Febiger, Philadelphia. It is written by Dr. Frederic A. Peeso, instructor in this subject in the University of Pennsylvania. Dr. Peeso is well known as the inventor of the split pin attachment for removable bridges, and for his technical skill as a bridge worker. As may be anticipated, the book is very largely based on his ideas of construction and design; at the same time a large amount of space is given to the hygienic requirements so frequently ignored in much of the present-day crown and bridge work. The author devotes much space to the preparation of roots and crowns to be used as abutments, and to the adjustment of occlusion, so that excessive stress may not be thrown on the abutment roots in articular function. The author makes a strong plea for his methods of design and construction and for more careful and scientific treatment of the anchoring teeth, and also for more skillful technical treatment. Contrary to the judgment of many able practitioners, the author advocates pulp extermination in most cases. The book contains one chapter devoted to the metallurgy involved, by Mr. Louis J. Weinstein, which adds materially to the worth of the book. Some of his statements in regard to alloys are new and interesting, especially in regard to the influence silver and copper have in making practicable alloys.

Frederick L. Ream and R. H. Riethmüller contribute a good chapter on the X-ray with reference to bridge work. The book is well made and the descriptions with the many illustrations make it a most valuable contribution on this subject.

GOULD AND PYLE'S CYCLOPEDIA OF MEDICINE AND SURGERY. This is the third edition of this work of ready reference to modern knowledge of the entire field of diagnosis and treatment. This edition is revised and edited by Dr. R. J. E. Scott and nearly a hundred other writers. The dental topics are presented by Dr. W. H. G. Logan of Chicago. His exposition of pyorrhœa alvolaris is good, but outline of treatment not as comprehensive as the gravity of the disease and the success in treatment at this date warrants.

Of course the work is intended to keep the medical man posted as to the newer and approved methods of treatment, but it will be of great value to the dental profession. In fact, it is like a whole medical library condensed to a single volume, which, although of rather large size, is readily handled. Every medical science topic is given adequate representation, and many of the articles are quite fully treated. A great many illustrations of the text material, as well as quite a few colored plates, make the descriptions clearer and the book less bulky than it would be. The work is published by P. Blakiston's Son & Co., Philadelphia, and the price is twelve dollars.

---

MOUTH HYGIENE. This is the publication of the lectures given by nineteen lecturers to the class in Oral Hygiene, conducted by Dr. Alfred C. Fones, in Bridgeport, Conn. The purpose of the course is to instruct women to administer oral prophylaxis to children in the public schools, people in institutions and to serve as assistants to dental practitioners. Dr. Fones has given the name dental hygienists to the persons completing the course of study satisfactorily. The lectures cover the following topics: General Anatomy, Special Anatomy, Physiology, Bacteriology, Inflammation, Deposits on the Teeth, Dental Caries, the Teeth, Malocclusion, Pyorrhœa

Alveolaris, Pathology, Dental Prophylaxis, Chemistry of Food, Dermatology, Personal Hygiene, Physical Hygiene, Public School Instruction in Oral Hygiene and work for Inmates of Public Institutions.

It should be expected that a book produced by so many persons and on such a variety of topics would lack somewhat in continuity and directness of statement, and perhaps this book may be somewhat unpedagogic for a text book. Nevertheless it is surprising that so excellent a book on an entirely new subject could be produced by this means and which would be of any great value either to the student or professional reader. Dr. Fones was exceedingly fortunate in securing excellent men to give his course of instruction, and the matter presented by them is scientific and well chosen; but, we can not help wondering if it was not so highly specialized and scientific as to be difficult for such students to assimilate. We are pleased, however, that the work was presented from the best scientific basis rather than to give it as a kindergarten course, or as a technical course with no scientific foundation. If the students absorbed the material given in this book and were able to formulate a system of personal technique based on it, we feel confident that the oral hygienist so trained will not only meet our need, but make a place of useful and profitable ministration in oral hygiene. The book is excellently printed and illustrated, and will be of much interest and value to every practitioner of dentistry. It would need considerable changing to make a college text book of it, but it could be read by any practitioner with profit and interest.

